# Fine-Scale Population Estimation by 3D Reconstruction of Urban Residential Buildings

**DOI:** 10.3390/s16101755

**Published:** 2016-10-21

**Authors:** Shixin Wang, Ye Tian, Yi Zhou, Wenliang Liu, Chenxi Lin

**Affiliations:** 1 National Engineering Center for Geoinformatics, Institute of Remote Sensing and Digital Earth, Chinese Academy of Science (CAS), Beijing 100101, China; wsx@irsa.ac.cn (S.W.); zhouyi@irsa.ac.cn (Y.Z.); liuwl@radi.ac.cn (W.L.); lincx01@radi.ac.cn (C.L.); 2 University of Chinese Academy of Science (UCAS), Beijing 100049, China

**Keywords:** fine-scale population estimation, morphological operations, ZY-3, dasymetric mapping, 3D model

## Abstract

Fine-scale population estimation is essential in emergency response and epidemiological applications as well as urban planning and management. However, representing populations in heterogeneous urban regions with a finer resolution is a challenge. This study aims to obtain fine-scale population distribution based on 3D reconstruction of urban residential buildings with morphological operations using optical high-resolution (HR) images from the Chinese No. 3 Resources Satellite (ZY-3). Specifically, the research area was first divided into three categories when dasymetric mapping was taken into consideration. The results demonstrate that the morphological building index (MBI) yielded better results than built-up presence index (PanTex) in building detection, and the morphological shadow index (MSI) outperformed color invariant indices (CIIT) in shadow extraction and height retrieval. Building extraction and height retrieval were then combined to reconstruct 3D models and to estimate population. Final results show that this approach is effective in fine-scale population estimation, with a mean relative error of 16.46% and an overall Relative Total Absolute Error (RATE) of 0.158. This study gives significant insights into fine-scale population estimation in complicated urban landscapes, when detailed 3D information of buildings is unavailable.

## 1. Introduction

Population estimations make indispensable contributions to the activities of organizations, businesses and governments, since the dispersion of energy and resources among different geographical regions is strongly dependent on the population size [1]. From an urban geographical perspective, Clark [2] initially studied monocentric models, where the population density was determined by the distance to the Central Business District (CBD), and proposed a negative exponential model with a constant gradient. Though other researchers, such as Newling [3] and Parr [4], improved this model by adding or modifying different parameters, Tobler [5] put forward that the exponential distance decay function was an approximation for the entire urban area, whereas its repeated use away from the urban center seemed unreasonable.

Undoubtedly, the negative exponential function was an empirical estimation [6]. Then, areal interpolations began to be realized by many scholars, such as Tobler [7], Lam [8] and Rase [9], who utilized census data as the model input to interpolate or disaggregate original data and obtained a refined population distribution surface. Besides, accuracy of methods in areal interpolation was largely improved when various ancillary data were incorporated, such as land use types, street networks and statistical surfaces, and one of the most representative models was the dasymetric mapping. Wright [10] performed binary divisions iteratively to disaggregate population density from general zones to detailed zones in Cape Cod (MA, USA) while keeping the volume preserved through a dasymetric model. However, Wright’s model was not easy to implement, therefore, Langford and Unwin [11] applied multivariable regression to compute population densities in dasymetric subzones. Other researchers such as Yuan et al. [12], Eicher and Brewer [13] and Mennis [14] classified land use into different types and redistributed census data among them. In addition, some researchers still aimed at further optimizing this method, which included Zandbergen and Ignizio [15] who compared the accuracy of different types of ancillary data used in dasymetric mapping; Nagle et al. [16] who represented and quantified the uncertainties in dasymetric modeling by the Penalized Maximum Entropy Dasymetric Model (P-MEDM); Stevens et al. [17] who produced a gridded population density at a 100 m spatial resolution through the Random Forest model. However, it was Mennis [18] who pointed out that the biggest challenge of dasymetric mapping was to develop standardized dasymetric mapping techniques.

Another approach commonly accepted by many researchers was statistical modeling, which was first proposed by Kraus [19]. To explore the relationship between population and remote sensed variables, there were usually six categories of ancillary datasets: urban areas (Tobler, [20]) including urban lights (Prosperie and Eyton [21]; Lo [22]; Zeng et al. [23]), land use (Kraus et al. [19]; Weber [24]; Langford and Harvey [25]; Lo [26]), dwelling units (Porter [27]; Collins and EI-Beik [28]; Lo and Chan [29]; Lo [30]), image pixel characteristics (Iisaka and Hegedus [31]; Lo [32]; Harvey [33]), impervious surface (Lu et al. [34] and Li and Weng [35]) and other physical or socioeconomic characteristics (Dobson et al., [36]; Liu and Clarke [37]; Balk et al. [38]). However, some problems have not been solved. Taking land use types as an example, the accuracy of population estimations was largely based on the detail of land use classes and the methods ignored the heterogeneity of population inside the same land use type. In addition, the spatial resolution of population estimation was also limited.

With the development of society, demographic information at finer resolutions had a significant impact on the economic, social, technological and humanitarian development of cities and is an indispensable component used in policymaking and planning [39]. Li and Weng [40] compared different ancillary data in getting fine-scale population estimations based on Landsat ETM+ imagery, and two conclusions were drawn: the land use-based method performed better than impervious surface and vegetation fractions; dasymetric mapping yielded better results than choropleth mapping. Leyk et al. [41] coupled spatial allocation procedures with a dasymetric model to allocate population to household microdata based on maximum entropy models, which refined the population distribution solution to a subtract level. However, a number of experiments demonstrated that land use data could not be used to conduct accurate population estimations at a fine scale [42]. Moreover, these methods were constrained by the selection of the spectral response variables or by a reliable validation during non-census years.

Considering that a large number of building units that are vertically stacked cannot be easily identified from 2-D photographs as only the roofs are visible, the height information is essential for the real structure of the buildings [43]. Besides, the 3D properties of urban buildings represent the three-dimensional nature of living space [44] and serve as essentially direct factors in estimating fine-scale populations. Wu et al. [45] used a deterministic model to estimate sub-block-level population through building volumes derived from geographic information system (GIS) building data and three housing statistics, and proposed a deterministic population estimation model relating with housing occupancy rate and average number of population per floor. However, there were some limitations that needed to be improved. For instance, researchers needed to know how to obtain housing statistics during intercensal years and how to get building footprints and volumes without extra model input. Alahmadi et al. [46] found that height information was helpful to improve the prediction accuracy compared with conventional population models. Qiu et al. [43] also adopted building volumes derived from LIDAR as an index to estimate population at the census block level. Xie et al. [47] estimated fine-scale population distribution from LiDAR-derived residential variables by a morphological algorithm and refining classification of residential buildings and realized that the height of buildings could be regarded as a crucial component for the corresponding models, but the cost of LiDAR datasets was high and periodical LiDAR data was unavailable. The coverage of specific areas needed to be assured ahead, then users can schedule fights to acquire data, which to a certain degree resulted in a lack of timeliness and cost-effectiveness.

To sum up, traditional population estimation models were time-consuming to conduct or subjected to the assumption that subzones were evenly distributed, which indicates they have limitations for fine-scale population estimation in heterogeneous urban regions [6]. Moreover, existing methods of estimating population in finer resolutions are subject to the availability of census data or the accuracy of classification methods. As for 3D building models, LIDAR data was mostly used but not timely to some degree. The above issues are supposed to be addressed appropriately. When the diversity and variability of urban buildings are taken into account by a majority of researchers, it is necessary to consider imagery with a finer resolution in order to improve the recognition [48], so HR imagery is necessary. Furthermore, morphological operations are appropriate for extracting features from HR images when spectral, texture, structure, scale and granularity are taken into account [49,50,51].

After considering all the factors listed before, this paper aims to acquire population distributions at a finer scale using HR satellite data (ZY-3) to reconstruct 3D information of urban residential buildings through morphological operations.

## 2. Study Area and Dataset

**Figure 1:**
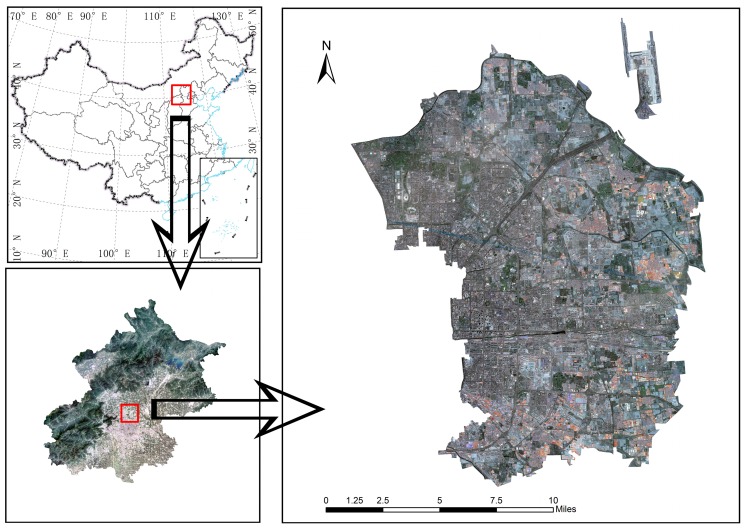
Location of the study area.

The study area is located in Chaoyang District, Beijing, which incorporates 42 administrative units covered by 10,153 × 13,295 pixels with a pixel size of 2.5 m, and serves as a typical example when the different population distribution from south to north due to unbalanced social and economic development is taken into account. Figure 1 indicates the specific location of the study area. Furthermore, numerous urban segments (e.g., buildings, roads, parking lots and a park) and undeveloped regions (e.g., bare soil, grasslands, and watersheds) are included. Though there are a variety of urban land use types, the research focused on residential buildings.

HR imagery is essential in the extraction of urban objects since most of them are noticeably smaller than natural features, and thus a significantly small pixel size is necessary for urban applications [52]. Accordingly, this experiment utilized two ZY-3 datasets (which principle parameters are listed in Table 1) obtained from the Satellite Surveying and Mapping Application Center (NASG), with a commonly Universal Transverse Mercator (UTM) coordinate system of 50N based on the WGS84 ellipsoid. The administrative map of Beijing at the county level for 2014 was obtained from the National Geomatics Center of China. Besides, validation data referred to the statistical yearbook downloaded from Beijing Chaoyang Statistical Information Net (http://www.chystats.gov.cn) in 2014. Furthermore, DSM was obtained from the National Administration of Survey, Mapping and Geoinformation, and point of interest (POI) data, which was collected from an urban digital map and incorporated five different types of buildings according to their utility (i.e., public services, financial buildings, commercial facilities, entertainment constructions and residential buildings), is also included since the location of buildings outperforms other ancillary datasets in population estimations [53].

## 3. Methodology

**Figure 2:**
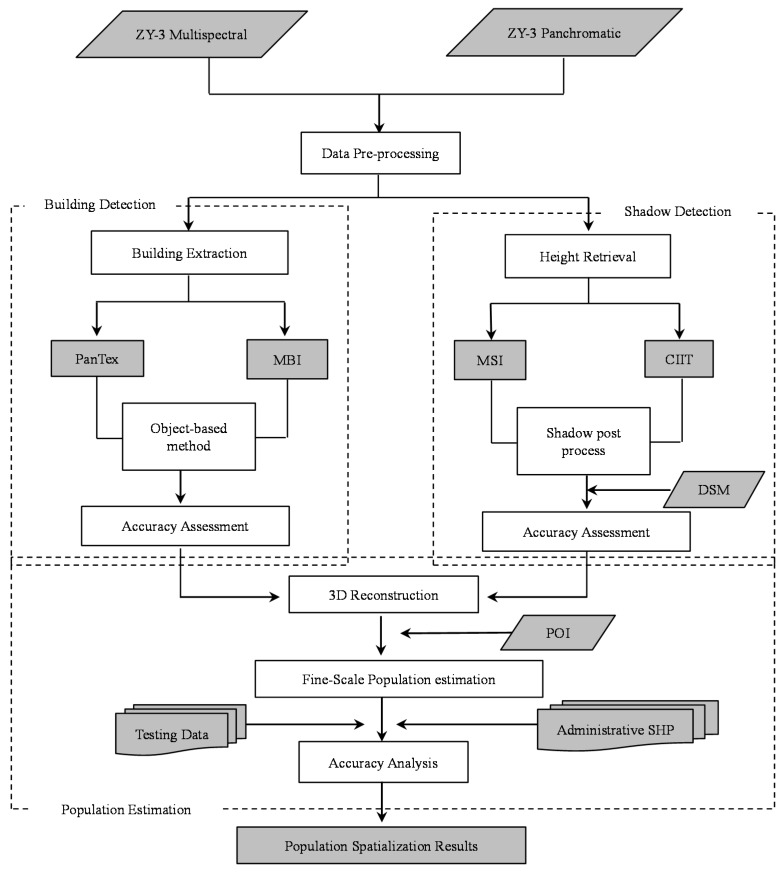
Flowchart of the research. Note: PanTex—Built-up Presence Index; MBI—Morphological Building Index; MSI—Morphological Shadow Index; CIIT—Color Invariant Indices; POI—Point of Interests; SHP—shapefile format processed by the ArcMap 10.2 software.

In this section, specific methodology pertaining to extracting 3D information of urban residential buildings and fine-scale population estimation would be illustrated. Detailed procedures are described in the flowchart shown in Figure 2.

### 3.1. Data Preprocessing

The preprocessing of data includes image ortho-rectification, registration, multispectral and panchromatic image fusion and extraction of the research area. Specifically, a rational polynomial coefficients (RPC) file, which utilized the sensor’s physical and orbit parameters along with appropriate ground control points to get the transform matrix, was firstly used for ortho-rectification of the ZY-3 datasets. As there were deviations between multispectral and panchromatic images, it was then crucial to register the image with higher spatial resolution (pan images). The Gram-Schmidt Spectral Sharpening algorithm was subsequently adopted for image fusion and finally the research area was extracted by clipping from regions of interests (ROIs). All of these processes could be accomplished automatically by the ENVI 5.1 platform.

### 3.2. Building Extraction

Various methods have been explored by scholars to detect urban buildings from high resolution or very high resolution (HR/VHR) images, particularly morphological operations [49,50,54]. Because of the high brightness in all visible bands and more evident textural features compared to many natural objects, morphological operations are appropriate for building extraction [55]. Furthermore, the object-based method is superior to the traditional per-pixel way in feature extraction [52], so it is reasonable to combine these two methods to extract building footprints in urban areas. In this step, two indexes (MBI and PanTex) are extracted through morphological operations and they are regarded as indispensable parameters for building extraction by the object-based method.

#### 3.2.1. PanTex Calculation

According to [54], PanTex was based on fuzzy rule-based composition of anisotropic textural co-occurrence measures through the gray-level co-occurrence matrix (GLCM). The foundation of the index depends on the fact that buildings cast shadows and thus produce high contrast in a local range. As a result, a rotation-invariant property of GLCM (i.e., contrast) is utilized to display such structural features of built-up areas. Then, PanTex is obtained by integrating different displacement vectors of the contrast by using a min operator in fuzzy set logic. Though the literature stated that five meters was sufficient for differentiating built-up and non-built-up areas, in this study, the PanTex is computed from the panchromatic image of ZY-3 after preprocessing with 2.5 m spatial resolution. In addition, given the distinction of image features in disparate regions, the results of PanTex derived from different moving window sizes are discussed latter in order to select the best result. The following steps were adopted:

Step (1) GLCM derivation

GLCM is a textural feature based on statistics summarizing the relative frequency distribution (i.e., how often one gray tone level will appear in a specified spatial relationship to another gray tone on the image [56]). In this study, ten different displacement vectors with distinct directions are selected to compute the GLCMs.

Step (2) Contrast measures extraction:

The textural measure of contrast is demonstrated as an effective characteristic to discriminate built-up and non-built-up areas. Consequently, contrast measure is adopted in this study and it is calculated using the following formulation:

(1) $$Contrast=\overset{n}{\underset{i=1}{\sum }}\overset{n}{\underset{j=1}{\sum }}{\left(i-j\right)}^{2}\times P_{i,j}$$

where *n* is the gray levels of the input image (in this case 256), and $P_{i,j}$ indicates the (*i*,*j*)th element of the GLCM. As there are ten different GLCMs, the contrast measure would produce ten results, respectively.

Step (3) PanTex construction:

Built-up areas are regions of the image where the textural contrast is high in all directions [54]. Thus, all textural features are integrated (i.e., ten contrast measures) by the min or fuzzy operator to obtain the PanTex:

(2) $$PanTex\left(i,j\right)=min\;(Contrast{\left(i,j\right)}_{m})$$

where *m* ranges from 1 to 10 indicating the ten contrast measures, and (*i*,*j*) means the position of the image pixel.

#### 3.2.2. MBI Calculation

The purpose of MBI is to relate the implicit properties of buildings (e.g., brightness, size, and contrast) to morphological features (e.g., reconstruction, granulometry and directionality) [49]. In order to find the best results of MBI, results based on several sizes of structural element are discussed latter. MBI is calculated and detailed procedures are described as follows:

Step (1) Top-hat reconstruction:

The difference between the original image and its morphological opening is defined as top-hat and white top-hat (*W_TH*) transformation, which is introduced as below:

(3) $$W\_TH\left(d,s\right)=b-\gamma_{b}^{re}\left(d,s\right)$$

where *b* is the maximum value among all multispectral bands and $\gamma_{b}^{re}$ is the result of opening-by-reconstruction from the brightness image. Besides, *s* and *d* represent the length and direction of a linear structural element (*SE*), respectively.

Step (2) Calculation of directional $\bar{W\_TH}$:

The multidirectional information of *W-TH* is acquired by averaging four directions of the *se*:

(4) $$\bar{W\_TH}\left(s\right)=\underset{d}{mean}\;W\_TH\left(s\right)$$

Step (3) Granulometry extraction:

Granulometry indicates the size and scale of an object in imagery. Accordingly, the differential morphological profile (DMP) is described as:

(5) $$DMP_{\bar{W\_TH}}\left(d,s\right)=\bar{W\_TH}\left(d,s+\Delta s\right)-\bar{W\_TH}\left(d,s\right)$$

where $s_{min}\leq s\leq s_{max}$ and Δs are the intervals of granulometry.

Step (4) Construction of MBI:

The MBI is defined as the average of $DMP_{\bar{W\_TH}}$:

(6) $$\{\begin{array}{c} MBI=\frac{\sum_{d,s}DMP_{\bar{W\_TH}}\left(d,s\right)}{D\times S}\\ S=\left(s_{max}-s_{min}\right)/\Delta s+1 \end{array}$$

where *D* and *S* represent the amount of directionality and the scale of profiles, respectively.

The specific method was illustrated in [50] where different parameters were selected according to the characteristics of the research area. In this experiment, four directions (0°, 45°, 90° and 135°) are chosen in Step 3 and this could get enough satisfactory results compared with more directions in the extraction of Directional $\bar{W\_TH}$ [49]. The size of *SE* is chosen according to the contextual and spatial characteristics of the image. Besides, MBI is constructed based on the phenomenon that buildings have larger values in $DMP_{\bar{W\_TH}}$ as they show higher local contrast in all four directions [50].

#### 3.2.3. Object-Based Method to Extract Buildings

According to the principle of the object-based method, detailed feature rules in this study are set after many trial-and-error tests with examinations of different combinations of parameters. The research chooses appropriate parameters (listed in Table 2) for building extraction through the PanTex and MBI methods after testing in a sample region shown latter, where scale, shape, and compactness are fixed parameters for segmentation. The previously extracted MBI is able to represent the main features of urban residential buildings; brightness is used to exclude low-reflective objects, such as water areas; NDVI could filter vegetation and length/width ratio is used to eliminate roads since roads have larger values of length/width ratio; rectangular fit is defined as the ratio between the number of pixels inside the rectangle which has the same area as the considered object, and the total number of pixels for the corresponding object [49]. Buildings have a large rectangular fit value. Shape index is obtained from the ratio between the perimeter and the square root of the area since buildings have smaller value of shape index than other objects [49]. After classification of buildings, a pixel-based object resizing algorithm is applied to fill small holes inside the building polygons. Each building’s area is computed through each of the segmentation object. These procedures are combined together to extract buildings from original images through the commercial software eCognition 9.0.

#### 3.2.4. Residential Building Refinement

In order to refine urban residential buildings within the study area, the last procedure is to exclude other objects (e.g., commercial buildings, overpasses, parking lots, and cemeteries). Therefore, POI is necessary to discriminate residential buildings from other irrelevant objects.

### 3.3. Height Retrieval

The height of buildings is computed by three main approaches: (i) multi-sensory images, such as aerial imagery and LIDAR data [57]; (ii) photogrammetry through stereo images [58,59,60]; (iii) calculation of shadow length or making a volumetric shadow analysis with the help of the geometrical relationships between the Sun and the satellite [61,62,63,64]. However, the cost and time requirements for the first way are much higher than the other two. Although it is possible to obtain the Digital Surface Model (DSM) of the research area by the second approach, the primary purpose of DSM is to describe overall topography on a large scale [65]. However, the main purpose of this research is to estimate individual building height, not the overall terrain, therefore, we chose the way based on the length of shadow to calculate building height.

Furthermore, the shadow extraction methods and their results on final building height estimation were not compared in previous literature. We further divide this method into two aspects: Morphological Shadow Index (MSI) and a transformation based on CIIT [66], and both of them are compared in this study. Though other approaches, such as HIS space transformation [67] and threshold segmentation based on the fact that hue values of shadowed regions is much bigger than their adjacent areas [68], were proposed, the final result shows large omission errors in shadow extraction and a considerable amount of disperse shadow speckles rather than connected shadow areas.

#### 3.3.1. Shadow Extraction

(1) MSI method

The approach for calculating MSI is similar to MBI, and the only difference is that we substitute white top-hat (*W_TH*) transformation into black top-hat (*B_TH*) transformation [50]:

(7) $$B\_TH\left(d,s\right)=b-\varphi_{b}^{re}\left(d,s\right)$$

where $\varphi_{b}^{re}$ is the result of closing-by-reconstruction from the brightness image. It is necessary to be aware that the high brightness of MSI indicates a higher probability of being shadows, which is exactly contrary to MBI.

(2) CIIT method

**Figure 3:**
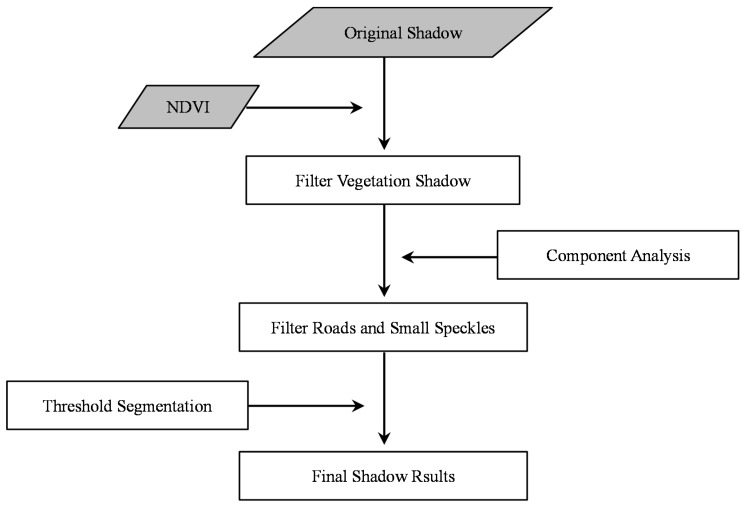
Shadow post-processing procedures.

Techniques of CIIT were computed according to [66] and only the third channel was used to identify the boundaries of shadows. The third index is:

(8) $$C_{3}=arctan\left(\frac{B}{max\left(R,G\right)}\right)$$

where *R*, *G* and *B* refers to the red, green and blue band of the original images. Then, a 3 × 3 texture-filter is applied to calculate the local variance of shadows around the neighborhood pixels. Though the primary goal in reference [66] was to detect the shadow boundaries and restore them, in this study, we applied the algorithm and acquired a continuous regions in which shadows are distributed with high brightness values. The shadows extracted by MSI or CIIT could not be used directly, and its post-processing steps are displayed in Figure 3.

It should be highlighted here that neither the MSI-based method nor CIIT consider the effect of vegetation and waters on shadow regions. In consequence, NDVI and NDWI should be added to filter the final results. Besides, image noises and many roads are particularly easy to classify as shadows. Accordingly, component analysis which describes the area and the length/width ratio within each component region is conducted to exclude small speckles and roads from real shadows. Both of these methods choose a threshold for final shadow extraction through the maximum between-class variance (MBCV) criterion, which believes that the threshold is chosen in order to maximize the separability between two modes that the histogram of an image should have [69]. This process is conducted in MATLAB 2014a. In addition, the shadows of non-residential buildings need to be removed with the assistance of POI.

#### 3.3.2. Shadow Length Calculation

According to the geometrical relationships between the sun and the satellite, the length of shadow could be decided through: (i) the solar altitude and azimuth angle, which are obtained from metadata of the satellite and they determine the angular relationship between buildings and their shadows; (ii) a series of parallel lines that can be plotted in accordance with the angle; (iii) the length of intersected part between original parallel lines and the shadow regions would be accepted as the final shadow length.

#### 3.3.3. Building Height Estimation

Considering that ZY-3 datasets are ortho-images, thus the building height can be computed as follows:

(9) H=L×tanβ

where β is the solar altitude (in this study is 68.68°), *L* is the length of a shadow and *H* is the corresponding building height.

### 3.4. Population Estimation

**Figure 4:**
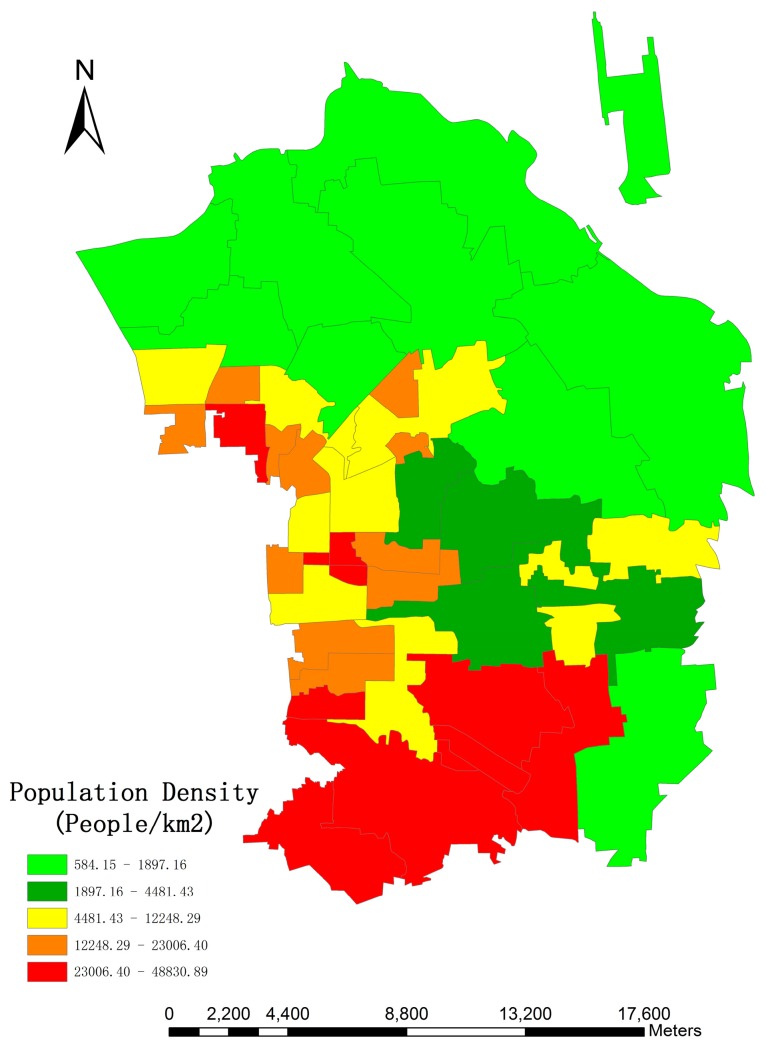
Population density in the research area.

**Figure 5:**
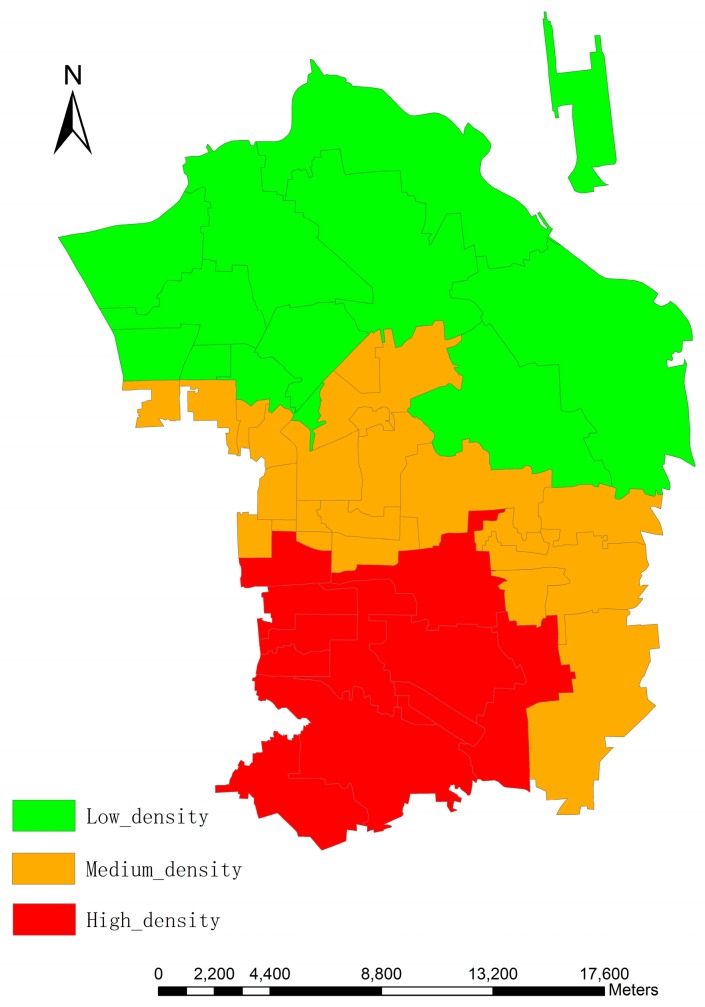
Dasymetric mapping of the population density in the research area.

Since dasymetric mapping is an effective and flexible method for estimating population, which minimizes the error within each dasymetric regions [18], this study divides the research area into three categories: high-density (15,448.06 people/km^2^), medium-density (7942.72 people/km^2^) and low-density (3060.53 people/km^2^), according to the population density calculated from original census data when the different living space per person and height per floor are considered. Figure 4 and Figure 5 show the distribution of population density and three dasymetric zones, respectively.

A mathematical relationship is built to estimate the fine-scale distribution of urban population. As a consequence, the population is estimated by:

(10) $$Pop_{i}=\frac{FS_{t}}{LA_{t}}\times \frac{BH_{t}}{AH_{t}}+C+\varepsilon_{t}$$

where *Pop_i_* indicates the population in building object *i*, *FS_t_* and *BH_t_* are the floor space and building height in zone *t*, respectively, and they could be calculated from each building object. *LA_t_* means living area per person and *AH_t_* is the height per floor in zone *t*. In order to meet the demands of the volume-preserving and pycnophylactic property coined by Tobler [7], *C* is the constant in the population model which is used to guarantee that the sum of population is equal to the statistical data in research area. $\varepsilon_{t}$ is the error correction term which is computed from least-square method based on field surveys.

In order to get *LA_t_* and *AH_t_*, we not only look for statistical books as a reference, but did field surveys by selecting samples from correspondent zones to get more accurate results. *LS_t_* could be computed by following formula:

(11) $$LA_{t}=\overset{n}{\underset{i=1}{\sum }}A_{i}/\overset{n}{\underset{i=1}{\sum }}P_{i}$$

where *A* is the living area, *P* is the correspondent population and *n* is the number of samples. Likewise, *AH_t_* can also be gotten by following equation:

(12) $$\;AH_{t}=\overset{n}{\underset{i=1}{\sum }}\left(H_{i}-RH_{i}\right)/\overset{n}{\underset{i=1}{\sum }}F_{i}$$

where *H*, *RH* and *F* indicate the total building height, correspondent roof height, and the number of building floors, respectively.

It is necessary to mention that, in this experiment, population value is displayed on each building objects not on the raster cells. Since population is estimated by the 3D information from each residential building, other irrelevant cells would be redistributed zero. However, the analysis of spatial distribution of population in research area would not be affected.

### 3.5. Accuracy Analysis

Since the 3D feature of buildings is reconstructed, three parts of accuracy analysis have to be included: building detection, height retrieval and population estimation. The accuracy results of the first two segments are used to find the optimal method in extraction of 3D information of buildings, and the population estimation result is used to validate the feasibility and reliability of our fine-scale population estimations using the proposed approach.

## 4. Comparison and Experimental Results

### 4.1. Building Extraction Results

**Figure 6:**
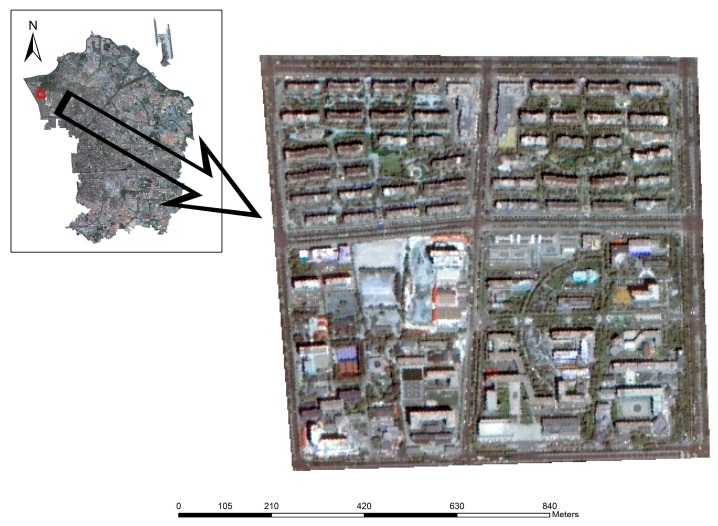
Location of the sample area.

Two approaches for building extraction based on morphological operations are compared in this study. In order to clearly display the effectiveness from different parameters in the building detection algorithm, one sample region is selected inside the research area (375 × 408 pixels) to show the results. Figure 6 shows the sample region in which quite a few research institutes, apartments and areas of vegetation are included.

**Figure 7:**
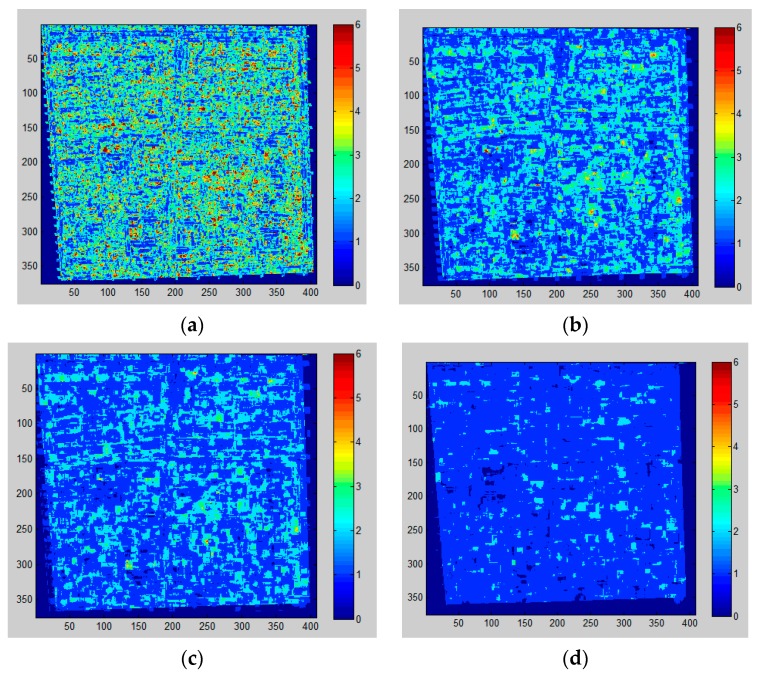
PanTex results derived from different moving window sizes. (**a**–**d**) indicate the results from window sizes of 4, 7, 9 and 14, respectively (a higher value means higher probability of buildings).

**Figure 8:**
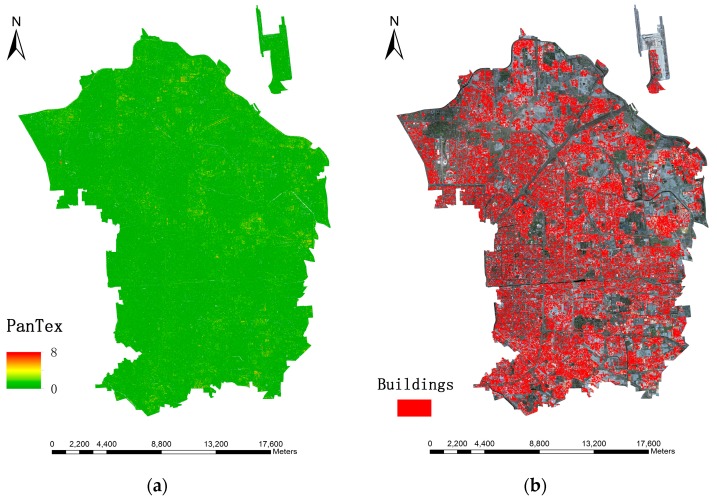
Building extraction results through PanTex. (**a**) PanTex in research area (window size = 7); (**b**) building extraction using object-based method (red regions symbolize buildings).

Figure 7 gives the PanTex result using different moving window sizes. As can be seen from Figure 7, the large window size (14) displays ambiguous information that causes problems in discriminating buildings from other objects, whereas the small window size (4) shows too many unnecessary speckles with an extremely slow speed. The medium window sizes (7 and 9) have comparative performance, whereas window size 7 is the more appropriate choice when the average physical size of building object (18 m) in sample area, integrality and separability of dwelling structure are taken into account. Figure 8 presents the PanTex results extracted from a moving window size of 7 and the final map of building extraction by object-based method.

**Figure 9:**
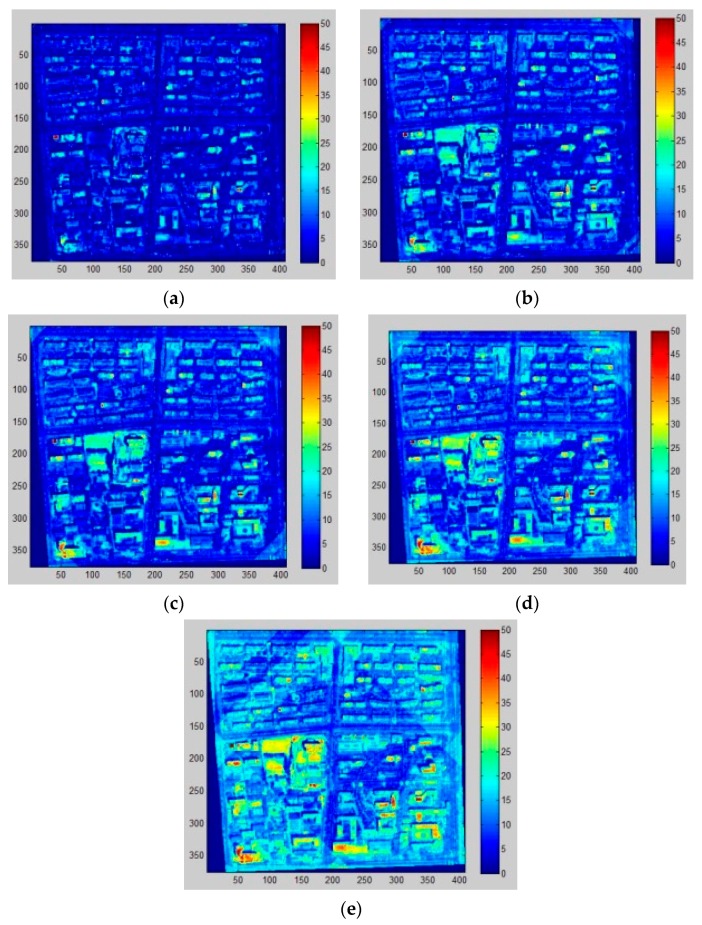
MBI result derived from different structural element sizes. (**a**–**e**) represent granulometry intervals (Δs) of 30, 15, 7, 5 and 2, respectively (a higher value means higher probability of buildings).

**Figure 10:**
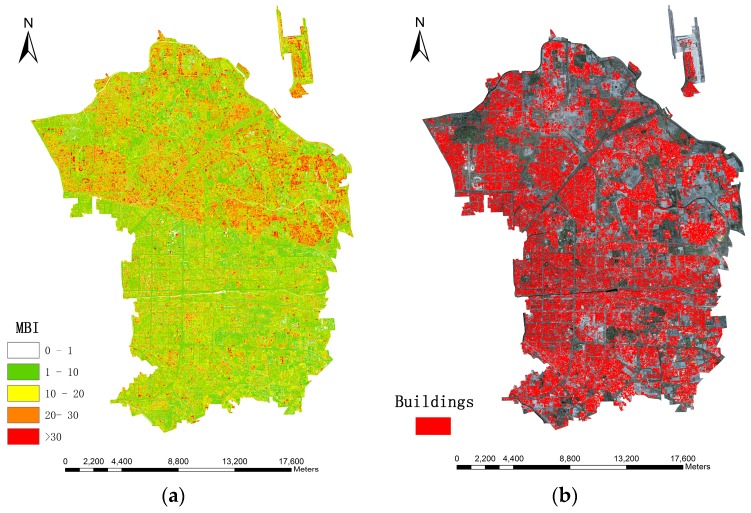
Building extraction results through MBI. (**a**) MBI of research area (Δs = 7); (**b**) building extraction by object-based method where red regions symbolize buildings.

Figure 9 shows the MBI results derived from distinct sizes of structural elements (*SEs*) in the same sample region. In this study, five intervals of the granulometry (Δs) are compared and Δs = 7 (i.e., *s* = [2, 9, 16, 23, 30, 37, 44, 51, 58, 65]) is selected as the best choice. To illustrate, Δs = 30 produces many stripes across the entire region, especially on the corners of the image, which is the trace of the *SEs* and this causes high-intensity area across a large area leading to poor detection of buildings. Δs = 2 contains large omission errors since a multitude of buildings displayed low intensity and they are more likely to be neglected in the following procedure. The remaining three (Δs = 5, 7 and 15) perform relatively well, showing fewer stripes on the corners and a marked contrast between buildings and other matters. Likewise, both physical size of building object, integrality and separability are the last standard to select, so Δs = 7 is the most appropriate choice. Figure 10 displays the MBI results of the research area and building extraction results through the object-based method.

Table 3 and Table 4 summarize the accuracy of building detection based on MBI and PanTex extraction through the object-based method, respectively. The validation points (574), which account for 70% of the total building objects, are randomly produced in the research area. According to these tables, the MBI method performs better in building extraction in urban landscapes with a higher overall accuracy and kappa coefficient (0.85 and 0.66) than the PanTex method (0.73 and 0.34). It was therefore adopted to refine the residential buildings in subsequent steps. Such a conclusion is in accordance with the literature [49]. Most of commission errors come from the roads, overpasses, shadows, parking lots, gazebos and open squares, which consist of similar materials to buildings and thus are difficult to clearly differentiate. The main omission error results from low buildings with small areas, and buildings surrounded by dense vegetation, which are more likely to be neglected by the algorithm.

Although buildings are almost all correctly detected, other irrelevant objects must be removed to improve the final accuracy of population spatialization. POI is adopted to precisely refine the residential buildings.

### 4.2. Height Retrieval Results

**Figure 11:**
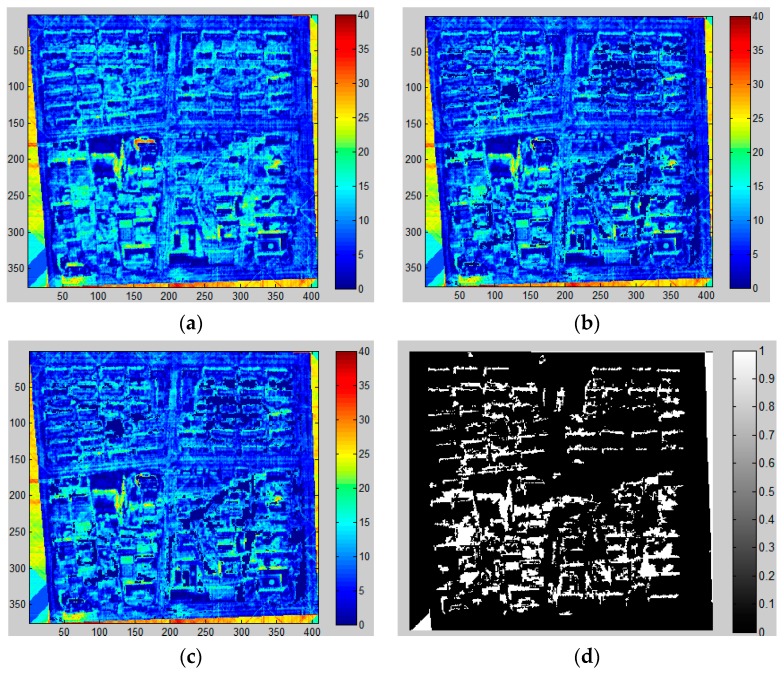
Shadow extraction using MSI based method in sample area. (**a**) MSI; (**b**) MSI filtered by NDVI and NDWI; (**c**) MSI after component analysis; (**d**) final shadow extraction results. (Higher value of MSI means higher probability of shadow in (**a**–**c**), the white color in (**d**) is the final shadow.)

**Figure 12:**
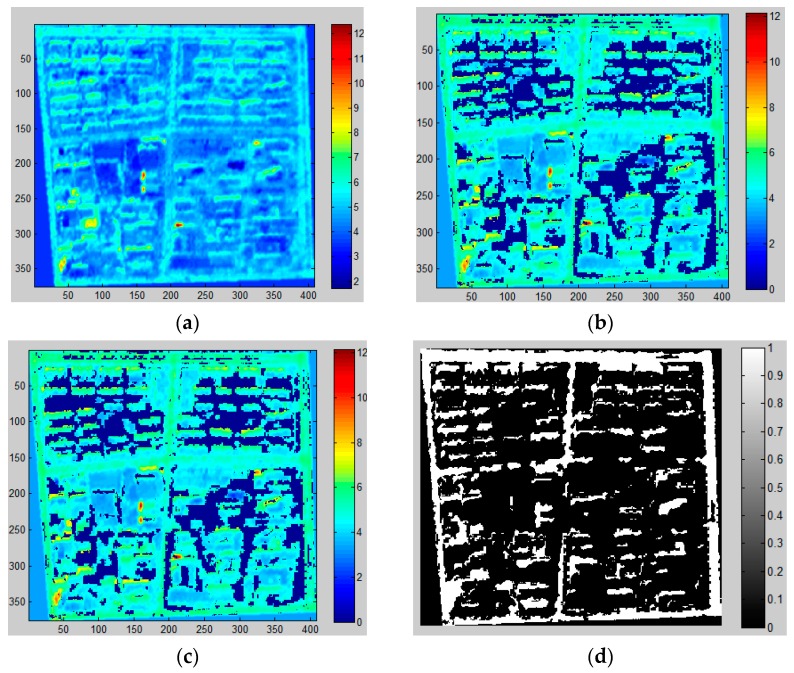
Shadow extraction using CIIT in sample area. (**a**) CIIT; (**b**) CIIT filtered by NDVI and NDWI; (**c**) CIIT after component analysis; (**d**) final shadow extraction results. (Higher value of CIIT means higher probability of shadow in (**a**–**c**), the white color in (**d**) is the final shadow.)

As stated before, two approaches are used to extract shadows in this study (i.e., MSI and CIIT). In order to display the change in an image during shadow extraction process, Figure 11 and Figure 12 demonstrate the procedures of shadow extraction and refinement based on MSI and CIIT in the sample area.

It can be observed from the above results that the MSI method performs better than CIIT in that the roads cannot be removed from the final results through the CIIT method and the shape of shadows extracted by the MSI method are more similar to true shadows (see Figure 6).

**Figure 13:**
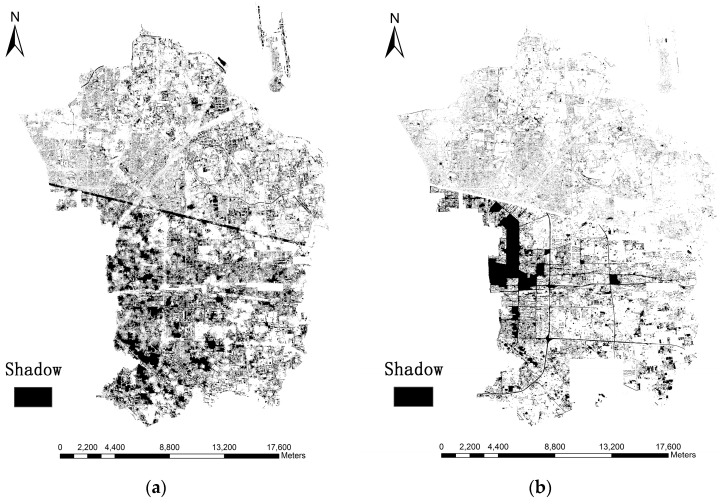
Results of final shadow extraction where black regions indicate shadows. (**a**) shadows extraced by the MSI method; (**b**) shadows extraced by the CIIT method.

Then the length of shadows is computed and building height is estimated. Specific procedures are described in Section 3. Figure 13 shows the shadows derived from the MSI method and CIIT method in the research area. It is reaffirmed that the MSI method generates a better result in shadow extraction than the CIIT method, especially in the western and southern parts of the research area, when the shape and purity of real shadows are taken into account.

**Figure 14:**
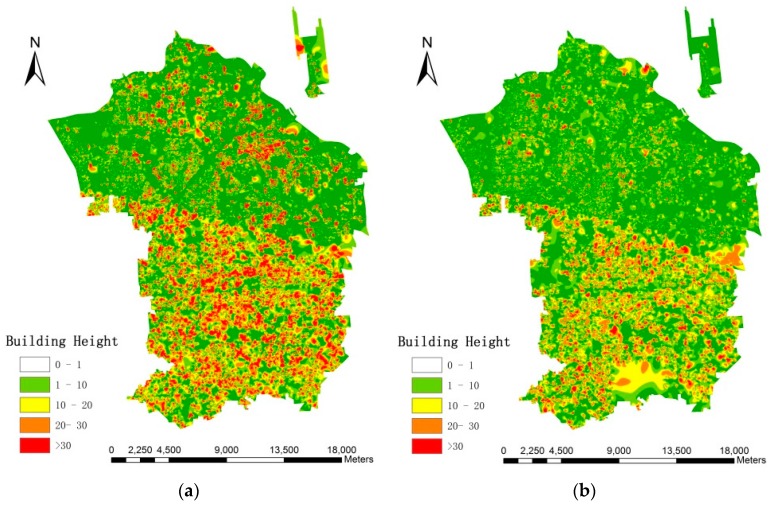
Results of building height using MSI and CIIT. (**a**) MSI method; (**b**) CIIT method.

Figure 14 displays the building height obtained with the MSI and CIIT approaches in the research area by calculating the length of shadow based on the geometrical relationship between the Sun and the satellite.

However, the location of shadow and residential buildings may not correspond well to each other given the complicated structure and distribution of buildings in urban landscapes. As a result, the height extracted from shadow length is utilized to interpolate the surface so as to compute the precise building height as much as possible. The results in this step are produced by the topo to raster tools in ArcMap 10.2. Likewise, the research randomly chooses 574 points produced from buildings to validate the height results using DSM as reference data. Table 5 gives the absolute error of using the MSI and CIIT methods. As can be seen from the Table 5, MSI performs overall better with less error than the CIIT method. Specifically, there are 25 points whose relative errors are bigger than 12.5 m in the CIIT method, whereas there are only seven such points in MSI. As for errors less than 2.5 m, 40.24% of the points meet the requirement in MSI compared with 36.24% in CIIT. Besides, the RMSE is 1.43 and 6.38 when using the MSI method and CIIT method, respectively.

### 4.3. Fine-Scale Population Spatialization Results

**Figure 15:**
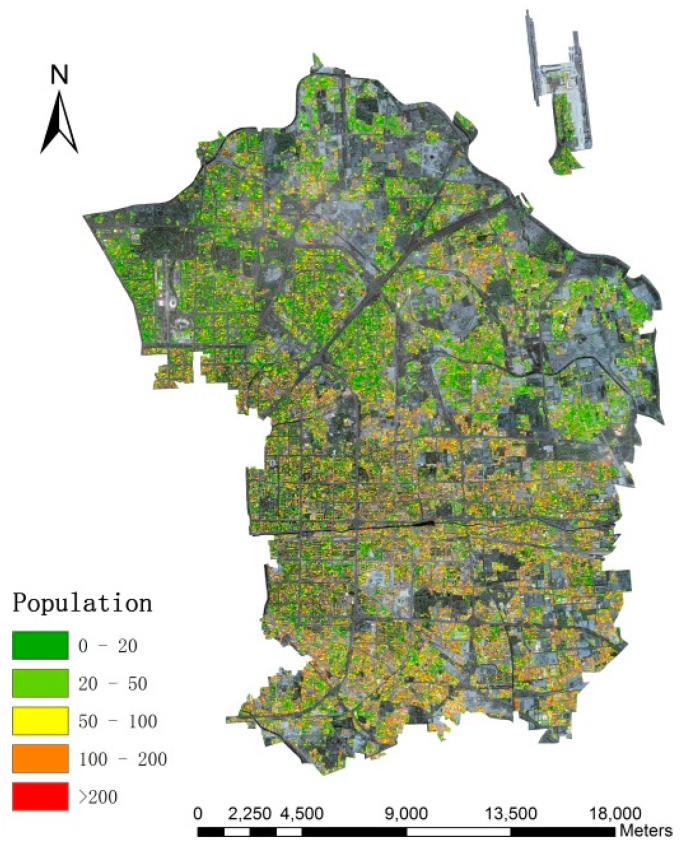
Population spatialization results (superimposed by the true color synthesis of ZY-3 imagery).

Since the MBI method performs better than PanTex in detecting building footprints and MSI outperforms the CIIT method in shadow detection and height retrieval, MBI and MSI are adopted to reconstruct a 3D model of residential buildings in this experiment. According to the model proposed in Section 3.4, the *LA* and *AH* are acquired from the different zones. The total population of Chaoyang in 2014 was 2,045,535, so we can calculate the constant *C* to fit the pycnophylactic properties. According to the model, we can obtain the population distribution in the research area. Final results are shown in Figure 15.

The image shows that people are gathered into blocks close to the transportation networks, which accords with the characteristics of urban citizen distribution. Besides, the southern part of research area displays an overall higher population density value than the northern part, which is attributed to the fact that many public parks (e.g., Olympic Forest Park) and non-residential buildings (e.g., National Swimming Center and Beijing National Indoor Stadium) are located in the north. Evidently, people are more willing to stay in southern part as it is closer to the central downtown area because of the biased social and economic development, which leads to superior economic development, greater job opportunities, and high-quality public services. For instance, there were 647 hospitals with 215,000 medical workers in total in 2013, and 438 hospitals with 171,000 relevant workers were located in the central downtown area, according to the survey of National Health and Family Planning Commission of the People’s Republic of China.

### 4.4. Accuracy Analysis

Table 6 summarizes the population results for all 42 administrative units in the research area (including the relative error, RE).

It can be seen from Table 6 that regions with a larger number of dwelling objects (No. 6, 8, 15, 19, 22 and 30) tend to have more population and usually lead to over-estimation errors. However, No. 33, which is located in the high-density zone with a relatively smaller value of *LA* and *AH*, is an exception and the model performed well in this region, which demonstrates that dasymetric modeling could improve the accuracy in this type of experiment.

**Figure 16:**
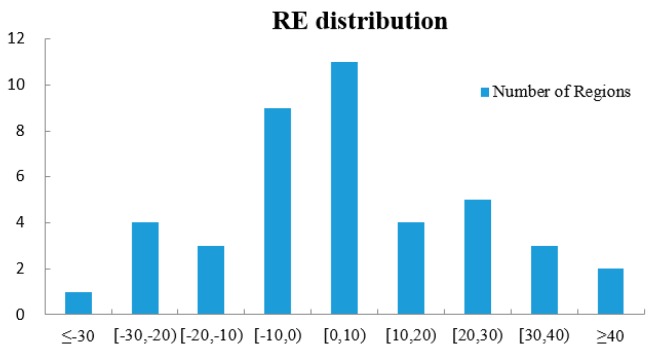
Accuracy analysis of fine-scale population estimation.

Figure 16 gives the relative error distribution in the research area. It can be seen that 20 regions (47.62%) yield very satisfactory results with an absolute value of RE of less than 10%. Besides, 27 regions (64.29%) produce reasonably good outcomes, with an absolute RE ranging from 10% to 20%. Furthermore, 17 regions (40.48%) show underestimation errors, with REs of less than 30%. This is attributed to the fact some shadows are shorter than the pixel size and a few of them may cast on buildings, which may not be detected by the algorithm. Besides, it cannot be ignored that there are two regions (No. 10 and 12) whose REs exceed 40%, and they are both over-estimation errors. The possible reason for these poorly performing regions is that they are both located in the boundary between high-density and medium-density zones, which has a higher degree of heterogeneity than others and leads to an inaccurate estimation of *LA* and AH. Although there exists inferior estimation in a few regions, the overall result, in general, is acceptable: 36 regions (85.72%) whose REs are less than 30% with a mean relative error of 16.46%.

From a technical point of view, the shape of building objects may not be identical to real buildings, which produces errors in calculating *FS*. As for estimating *BH*, it is closely related with the conditions of shadows as we will explain in the next section. Besides, high-density areas tend to have lower values of *LA* and *AH* and thus are more likely to result in over-estimation errors when high building densities are considered. Moreover, *LA* and *AH* in each zone are estimated by samples, so the possibility of sampling errors cannot be ignored either. In addition, *FS* and *BH* are extracted from MBI and MSI. Such errors could be propagated to the final population estimation, since not each building could be accurately detected by the threshold segmentation of MBI and height could not be estimated without any errors through MSI and shadow length calculation either.

**Figure 17:**
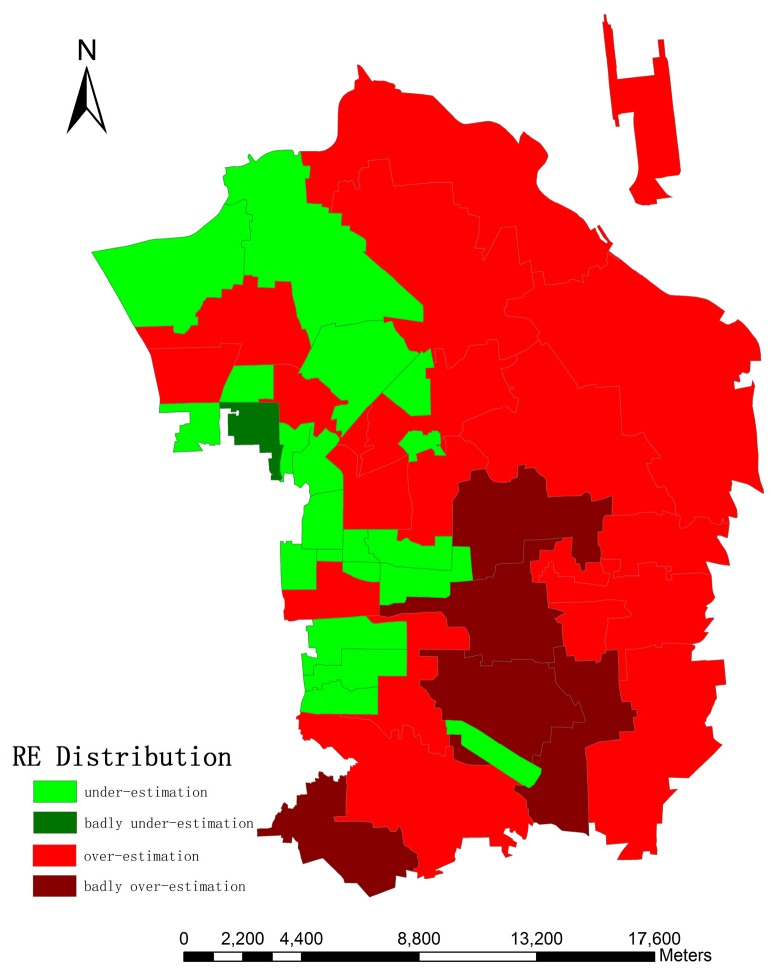
Spatial distribution of RE in the research area (original one has been changed; bad regions indicate RE > 30%).

Figure 17 gives the spatial distribution of RE throughout the research area. According to Table 6, 25 over-estimated regions are distributed in the eastern part, which can be explained by the fact that the building density in the east is relatively higher than in the west in the research area, which leads to a high density of shadows and an over-estimated number of buildings and more positive errors in height retrieval and building detection. As for the western parts, there are more public parks that contain larger areas of vegetation and more bare land than in the eastern areas, which gives rise to a larger omission error in the extraction of building footprints. Obviously, there is one badly under-estimated region in the northwest part. It can be explained by the reason that this region located in the intersection of different population density maps as stated before and these regions may have errors in estimation of *LA* and *AH* because of their internal heterogeneity. Likewise, three of four bad over-estimation errors are also in this situation. Another one (No. 40 in Table 6) is close to the central downtown of Beijing (i.e., Dongcheng District), whose building density is extremely high, which leads to positive errors like we stated before.

Total Absolute Error (TAE) is more robust for skewed distribution of population than Root Mean Square Error (RMSE) [70], and besides, *RTAE* (*RTAE* = *TAE/P_total_*,) are adopted to further analyze the model accuracy when a better reading is taken into consideration. *RTAE* is calculated as follows:

(13) $$RTAE=\frac{\sum \left|P_{i}-P_{j}\right|}{\sum P_{j}}$$

where *P_i_* and *P_j_* indicate the model value and statistical value of population in each subunit, respectively. The *RTAE* could be computed based on Table 6, and the result is 0.158. According to the definition of *RTAE*, a value of 0 means a perfect estimation, whereas a *RTAE* of 2 means a completely wrong estimation, so this value shows really reliable performance.

## 5. Discussion

In this paper, we proposed the idea that fine-scale population distribution could be estimated by 3D reconstruction of urban residential buildings through building detection and height retrieval with HR images. Specifically, we compared the methods of building detection through two morphological operations (i.e., MBI and PanTex) in large heterogeneous urban regions, and the final results demonstrate that MBI outperforms the PanTex method. Such comparisons are essential in choosing the most appropriate morphological index when researchers decide to extract building footprints. Besides, shadow is a unique characteristic that was easily ignored before, but it has been of concern to most researchers in the current state with the development of HR images. In this experiment, MSI and CIIT were compared in shadow extraction and building height retrieval, which has not been done before, and this provides an innovative way to extract 3D information without heavy field surveys. Moreover, this research combined building detection and height retrieval to reconstruct 3D information of residential buildings to estimate fine-scale population, which has not been researched so far, and it produces reasonable results. In the population estimation process, dasymetric mapping models are successfully incorporated by dividing the research area into different density regions, and such step greatly improves the accuracy of parameters (*LA* and *AH*) estimation and population distribution.

However, the research cannot ignore the errors that are produced during the entire process. There mainly refer to three aspects, summarized as follows:

(1) Building extraction errors

It is difficult to acquire the dimensions of densely spaced buildings in a heterogeneous area when the fixed parameters of algorithms are taken into account. In addition, roads, bare soil, and open areas are hard to distinguish from buildings as they have similar textural and spectral features. It may be more accurate to segment different regions with different combinations of parameters.

(2) Shadow detection errors

Several errors in shadow extraction may occur: (i) there is no shadow around some buildings due to the fact that the Sun’s angle was near the nadir when the image was acquired; (ii) dense buildings lead to dense shadows, which are gathered as large dark patches and bring about errors in calculation of shadow length; (iii) shadows could not be accurately identified everywhere, so the number of buildings and shadows may not be consistent in the same district; (iv) it is hard to accurately distinguish between the shadows of vegetation, buildings and other tall objects; (v) shadows of buildings with regular shapes (e.g., cuboid and parallelepiped) are easily identifiable, but irregular shadows cast from various building shapes are hard to capture accurately; (vi) the accuracy of shadow detection is influenced by the local surface slope in the research area [63].

It is noted that the largest positive height error occurs when the buildings are densely distributed and thus shadows are merged together, as previously discussed, which results in longer shadow lengths. Likewise, low buildings with small area are easily neglected and generate negative errors. As a result, it is easier to identify shadows separated from each other with complete and regular shape. However, the shadow of ZY-3 is relatively shorter when the images were acquired and many possible errors could be avoided, such as gather problems and shadows casting on buildings.

(3) Population estimation errors

The first type of estimation error may relate to housing occupancy rate. Specifically, newly developed regions, such as the northeastern part of the research area, with a fast growth rate may contain many multi-floored apartments, and their occupancy rate is relatively low. On the other hand, old citizens might have small houses with large yards resulting in high occupancy rates [45]. Furthermore, census data are de jure population reports which survey all usual residents in the given region, regardless of whether they are physically present there at the certain date [6]. In addition, some citizens living underground cannot be detected in such a way.

## 6. Conclusions

We believe that the principal outcome of our work lies with the following three aspects: (i) high resolution images was utilized to reconstruct 3D model of residential buildings through morphological operations; (ii) different methods of shadow extraction based on ZY-3 images and their final impacts on building height retrieval were compared; (iii) fine-scale population estimation was achieved by 3D reconstruction of urban residential buildings, and a deterministic model in a relatively large scope through a more feasible approach was proposed. This method does not need the classification of land use types for the model input and final result shows great potential in determining urban citizen distributions at finer resolutions in the future.

Though the errors are propagated from one step to the next, the overall accuracy within a relatively large and complicated urban area is promising, with a mean relative error of 16.46% and RTAE of 0.158. Frankly speaking, not much more can be expected at this early stage since morphological indexes derived from the remote sensing techniques are probability distributions of buildings and shadows, but it is a significant start to exploring the potential of using the spectral, spatial and textural information of HR images. Besides, inherent uncertainties of ancillary variables also exist, as stated in previous research [46]. However, it still demonstrates that fine-scale population estimation could be connected well with reconstructing 3D features of residential buildings.

Considering that POI was utilized as ancillary data for the model input, further research would focus on finding a more accurate and fast method for residential building refinement by combining the detailed spectral or textural characteristics of the images. Furthermore, we hope to classify residential buildings into several categories (e.g., single-family dwelling, multi-family dwelling and other types) based on the properties of citizens (e.g., income, age and education) and environmental factors (e.g., green area per capital and transportation accessibility). In addition, the correlation of population density with other factors, such as building density, accessibility of transportation networks, GDP and supporting capability of environmental resources, would be further analyzed in urban landscapes based on empirical sampling, regression analysis and other relevant approaches. Finally, we also intend to study and analyze the dynamics of population migration in an urban environment with a cellular automata model which is useful to simulate the mobility of urban citizens [71].

## Figures and Tables

**Table 1:** Parameters of the ZY-3 dataset.

Camera Mode	Panchromatic (pan)
	Multispectral (mux)
Spatial resolution	pan: 2.1 m, mux: 5.8 m
Wavelength (nm)	pan: 450–800
	mux: blue: 450–520, green: 520–590, red: 630–690, NIR: 770–890
Image width (km)	pan: 50 × 50, mux: 52 × 52
Cloud-cover degree	0%
Orbit number	18,862

**Table 2:** Parameters set for building extraction by the object-based method.

Procedure	PanTex	MBI
Multiresolution segmentation	scale: 25	
	shape: 0.6	
	compactness: 0.4	
Classification	PanTex ≥ 1.45	MBI ≥ 6
	brightness ≥ 136	
	NDVI < 0.1	
	1.5 ≤ length/width ratio ≤ 4.5	
	rectangular fit ≥ 0.6	
	shape index ≤ 2.6	
Post-processing	reference: object	
	mode: growing	
	value: 0.5	
	box size in X and Y: 5	

**Table 3:** Accuracy of MBI methodology.

User Class/Sample	Buildings	Non-Buildings	Sum
Confusion Matrix			
Buildings	158	57	215
Non-buildings	29	330	359
Sum	187	387	
Accuracy			
Producer	0.85	0.85	
User	0.73	0.92	
Kappa per class	0.75	0.86	
Totals			
Overall Accuracy	0.85		
Kappa	0.66		

**Table 4:** Accuracy of PanTex methodology.

User Class/Sample	Buildings	Non-Buildings	Sum
Confusion Matrix			
Buildings	86	55	141
Non-buildings	100	333	433
Sum	186	388	
Accuracy			
Producer	0.46	0.86	
User	0.61	0.77	
Kappa per class	0.29	0.43	
Totals			
Overall accuracy	0.73		
Kappa	0.34		

**Table 5:** Error distribution of height retrieval from MSI and CIIT.

Absolute Error (m)	CIIT	Percentage (%)	MSI	Percentage (%)
≥12.5	25	4.36	7	1.22
[10.0, 12.5)	58	10.10	38	6.62
[7.5, 10.0)	66	11.50	52	9.06
[5.0, 7.5)	93	16.20	101	17.60
[2.5, 5.0)	124	21.60	145	25.26
[0, 2.5)	208	36.24	231	40.24
Total	574	100	574	100

**Table 6:** Accuracy analysis of fine-scale population estimation.

No.	Region Name	Population (Statistics)	Dwelling Objects	Population (Model)	RE (%)
1	Anzhen Sub-district	48,467	711	46,031.227	−5.03
2	Olympic Village Sub-district	57,509	1771	55,101.244	−4.19
3	Changying Sub-district	24,770	1240	32,120.220	+29.67
4	Chaowai Sub-district	43,162	496	39,569.780	−8.32
5	Chaoyangbalizhuang Sub-district	85,897	1049	61,229.257	−28.72
6	Cuigezhuang Sub-district	24,448	4691	30,771.512	+25.87
7	Datun Sub-district	71,615	2345	77,291.336	+7.93
8	Dongba Village	37,907	4732	38,612.750	+1.86
9	Dongfeng Sub-district	30,926	1187	37,596.391	+21.57
10	Dougezhuang Village	12,719	2178	18,841.690	+48.14
11	Fatou Sub-district	31,964	735	29,591.992	−7.42
12	Gaobeidian District	48,234	2969	71,468.861	+48.17
13	Guanzhuang Sub-district	57,269	2853	73,120.667	+27.68
14	Hepingjie Sub-district	98,710	881	59,449.281	−39.77
15	Heizhuanghu Village	49,681	3806	51,199.561	+3.06
16	Hujialou Sub-district	64,264	416	49,410.197	−23.11
17	Jianwai Sub-district	42,302	1265	54,101.568	+27.89
18	Jiangtai Sub-district	26,119	2200	28,267.330	+8.23
19	Jianzhan Sub-district	59,144	6891	60,010.514	+1.47
20	Jingsong Sub-district	73,200	1003	59,991.594	−18.04
21	Jiuxianqiao Sub-district	69,146	1297	70,233.239	+1.57
22	Laiguangying Sub-district	47,269	3889	51,471.291	+8.89
23	Liulitun Sub-district	62,168	891	48,996.313	−21.19
24	Maizidian Sub-district	21,674	954	24,664.127	+13.80
25	Nanmofang Sub-district	59,499	2014	70,155.476	+17.91
26	Panjiayuan Sub-district	81,055	762	59,981.261	−26.00
27	Pingfang Sub-district	33,492	2673	46,781.497	+39.08
28	Sanjianfang Sub-district	71,011	1443	78,501.201	+10.55
29	Sanlitun Sub-district	38,285	728	36,799.497	−3.88
30	Shibalidian Sub-district	38,950	6658	41,719.462	+7.11
31	Beijing Airport Sub-district	24,735	832	25,184.881	+1.82
32	Shuangjing Sub-district	72,360	1063	66,849.199	−7.62
33	Sunhe Sub-district	21,979	3760	26,109.591	+18.79
34	Taiyanggong Sub-district	45,381	1147	48,487.497	+6.85
35	Tuanjiehu Sub-district	36,869	264	33,996.495	−7.79
36	Wangsiying Village	19,517	2394	27,106.487	+38.89
37	Wangjing Sub-district	82,815	2321	82,599.481	−0.26
38	Xiangheyuan Sub-district	34,636	350	32,841.498	−5.18
39	Xiaoguan Sub-district	51,401	663	45,998.869	−10.51
40	Xiaohongmen Sub-district	29,112	2100	40,009.487	+37.43
41	Yayuncun Sub-district	48,858	858	53,299.819	+9.09
42	Zuojiazhuang Sub-district	67,016	799	59,996.794	−10.47
	Total	2,045,535	81,279	2,045,560.434	-

Where ‘+’ and ‘−’ represent over-estimation and under-estimation, respectively.
